# Rate of lumbar paravertebral muscle fat infiltration versus spinal degeneration in asymptomatic populations: an age-aggregated cross-sectional simulation study

**DOI:** 10.1186/s13013-016-0080-0

**Published:** 2016-08-05

**Authors:** Rebecca J. Crawford, Thomas Volken, Stephanie Valentin, Markus Melloh, James M Elliott

**Affiliations:** 1 Institute for Health Sciences, School of Health Professions, ZHAW, Zurich University of Applied Sciences, Technikumstrasse 81, CH-8401 Winterthur, Switzerland; 2 Faculty of Health Sciences, Curtin University, Perth, Australia; 3 Equine Clinic, University of Veterinary Medicine, Vienna, Austria; 4 Institute for Sport, Physical Education & Health Sciences, The University of Edinburgh, Edinburgh, UK; 5 Centre for Medical Research, University of Western Australia, Perth, Australia; 6 Feinberg School of Medicine, Northwestern University, Chicago, USA; 7 School of Health and Rehabilitation Sciences, University of Queensland, Brisbane, Australia

**Keywords:** Lumbar spine, Paravertebral muscles, Ageing, Multifidus, Erector spinae, Psoas, Disc degeneration, Asymptomatic adults, Magnetic resonance imaging, Fatty infiltration

## Abstract

**Background:**

The spinal column including its vertebrae and disks has been well examined and extensively reported in relation to age-aggregated degeneration. In contrast, paravertebral muscles are poorly represented in describing normative degeneration. Increasing evidence points to the importance of paravertebral muscle quality in low back health, and their potential as a modifiable factor in low back pain (LBP). Studies examining normative decline of paravertebral muscles are needed to advance the field’s etiological understanding. With a novel approach and based on published data, we establish and compare decline rates of imaging features for degeneration of lumbar vertebrae and disks, versus fatty infiltration in paravertebral muscles in asymptomatic adults.

**Methods:**

Our cross-sectional simulation study examined age-aggregated data from three published studies who reported on asymptomatic adults spanning 18–60 years. Prevalence rates of imaging degenerative features of the spinal column were examined via logistic regression and compared with percentage fatty infiltration in erector spinae, multifidus and psoas using synthetic data and Monte Carlo simulation with 10,000 endpoint-specific regression iterations. General linear regression models were employed to estimate marginal effects of age reported as a one-year change rate (with 95 % confidence intervals) for comparisons between all reported spinal features.

**Results:**

Declines in multifidus (0.24 & 0.11 %/year), erector spinae (0.13 & 0.07 %/year), and psoas (0.04 %/year) occur at similarly slow rates to disk protrusion (0.25 %/year), annular fissure (0.15 %/year), and spondylolisthesis (0.29 %/year). Multifidus showed a trend for faster decline than erector spinae, particularly in men. Of the features examined, disk signal loss declined fastest, and psoas muscle the slowest.

**Conclusions:**

Degeneration of lumbar paravertebral muscles occurs slowly in asymptomatic adults, with a tendency to be most pronounced in multifidus. Rate of decline of spinal structures represents a novel variable that warrants inclusion as a known feature of the expected degenerative cascade, and to provide a basis for comparison to diseases of the spine in research and clinical practice. Concurrent examination of spinal features using advanced imaging to improve muscle analysis would be a strong addition to the field.

## Background

Low back pain (LBP) causes more global disability and lost healthy years than any other condition [1]. It is a common health problem forecast to have a wider societal impact alongside an increasingly ageing population [2]. Identifying modifiable risk factors associated with LBP is important in influencing the disease, and necessary in understanding its etiology to develop targeted and effective interventions. While LBP is a multifactorial condition, lumbar paravertebral muscles are receiving increased attention for their therapeutic, diagnostic and prognostic (referred to as ‘theranostic’) potential.

Imaging features of spinal degeneration that are associated with LBP are also prevalent in asymptomatic individuals [3]. Determining the natural history of ageing is fundamental in understanding the spine in both health and disease. Despite an established association between paravertebral muscle degeneration and LBP [4–10], only a modest literature describes the role of these muscles in normal ageing [11, 12]. The importance of paravertebral muscles in optimizing back health is increasingly acknowledged and investigations determining normative degenerative change and muscles’ capacity to influence the course of recovery of LBP are needed.

In their meta-analysis of imaging studies, Brinjikji and colleagues [3] describe the prevalence of spinal column degenerative features in asymptomatic adult spines according to decade, but without reference to muscle. This omission likely reflects the paucity of available literature in describing normal (or asymptomatic) ageing of paravertebral muscles [11–13], and thus a relative lack of recognition towards non-invasive and reproducible quantitative measurement of soft-tissues exists in clinical practice. Traditional studies examining spinal muscle quality in LBP have employed qualitative grading based on the method of Kjaer et al. [7], or various semi-quantitative and quantitative methods that determine fat proportion within a cross-sectional area and defined region of interest based on counting pixel number and signal intensity [4, 14, 15]. As an advance from this practice, multi-echo imaging techniques like Dixon [16, 17] and proton-density methods [18] are, or should be, considered for contemporary studies as quantification of skeletal muscle fat content based on these imaging sequences is shown to have superior accuracy, visualization, speed of acquisition and utility [5, 19–23]. Comparing the wealth of literature reporting the natural history of degeneration of the vertebra and disks, with the recently promoted quantification studies describing muscle change is therefore somewhat difficult.

Fatty infiltration (FI) in skeletal muscle is an accepted feature of declining muscle structure and quality [22, 24]. While studies have identified increasing paravertebral FI with age in volunteers with [6, 25–27] and without [11–13] LBP, its etiology in normative decline, and relationship to other features of spinal degeneration, is poorly understood. Divergent from an earlier understanding, Hodges et al. [28] describe muscle FI as a feature of structural remodeling rather than muscle atrophy in the ovine multifidus (MF) after experimental and controlled injury involving the intervertebral disc. Human lumbar paravertebral muscle fat content has been associated with spinal features of degeneration (e.g. facet joint osteoarthritis [6], spondylolisthesis [6], disc narrowing [6, 9], and type 2 Modic change [9]). However, further information regarding the relationship between normative declining muscle quality and other features of spinal degeneration is necessary to better understand their combined impact, causation and potential for change with clinically-relevant and more informed interventions.

In this cross-sectional study, we use a novel simulation approach to compare age-related degenerative imaging features of the spinal column and paravertebral muscle FI of asymptomatic volunteers as documented in three published studies: Brinjikji et al. [3] describe age-specific prevalence rates of spinal column degeneration based on a meta-analysis of 33 imaging studies reporting across seven decades of life (20–89) [3]; Valentin et al. [12] report percentage of FI for psoas major (psoas), MF and erector spinae (ES) comparing two age-groups (18–25 with 45–60 years); and Crawford et al. [11] report FI in MF and ES across four decades (20–29, 30–39, 40–49, 50–60 years). Both muscle studies employed a standard multi-echo MRI sequencing that has been validated against biopsy and across magnetic fields [29]. Our primary aim was to determine a yearly rate of decline of muscle tissue (defined as increased percent FI) as compared with reported degenerative changes of the spinal column, given the latter typically benchmarks the natural history of age-related spinal change.

## Methods

To enhance comparability between the studies, we included published data for each study that represented subjects between 18 and 60 years. Based on published age-aggregated information (Table 1), we determined the marginal effect of age with corresponding 95 % confidence intervals (CI) for each degenerative feature as follows: For the Brinjikji et al. [3] study that reported prevalence rates for eight degenerative features, a sample of observations for each of the degenerative features corresponding to the summed total of subjects in the first four age groups from their study was created. All statistical analyses, including simulations, were carried out in Stata 14 (StataCorp, College Station, TX).

**Table 1:** Reference data used for age-specific prevalence estimates (%) for degenerative features of the spinal column (Brinjikji et al. [3]) and paravertebral muscle fat (%; ± standard deviation; Crawford et al. [11] and Valentin et al. [12]) according to age. Sample sizes are in parentheses

Study		Age (years)				
Brinjikji et al. 2015 [3]	Imaging Finding	20	30	40	50	*n*
	Disk degeneration	37 % (273)	52 % (604)	68 % (415)	80 % (311)	(1603)
	Disk signal loss	17 % (46)	33 % (142)	54 % (352)	73 % (73)	(613)
	Disk height loss	24 % (15)	34 % (163)	45 % (186)	56 % (208)	(572)
	Disk bulge	30 % (55)	40 % (101)	50 % (151)	60 % (123)	(430)
	Disk protrusion	29 % (87)	31 % (468)	33 % (490)	36 % (363)	(1408)
	Annular fissure	19 % (167)	20 % (350)	22 % (426)	23 % (53)	(996)
	Facet degeneration	4 % (0), (100)^a^	9 % (0), (100)^a^	18 % (596)	32 % (53)	(849)
	Spondylolisthesis	3 % (0), (100)^a^	5 % (0), (100)^a^	8 % (31)	14 % (53)	(284)
Crawford et al. 2015 [11]	Paravertebral muscles	20–29	30–39	40–49	50–60	
	MF + ES	12.4 ± 3.7(20)	15.9 ± 5.0(20)	15.7 ± 4.5 (20)	18.1 ± 7.8 (20)	(80)
	Multifidus	16.3 ± 5.3 (20)	20.8 ± 6.4 (20)	21.6 ± 6.1 (20)	24.1 ± 8.7 (20)	(80)
	Erector spinae	10.1 ± 4.7 (20)	12.9 ± 4.3 (20)	12.4 ± 3.4 (20)	14.7 ± 7.3 (20)	(80)
Valentin et al. 2015 [12]	Paravertebral muscles	18–25		45–60		
	Psoas	35.7 ± 2.0 (12)		37.1 ± 1.5 (12)		(24)
	MF + ES	40.2 ± 3.0 (12)		42.8 ± 2.9 (12)		(24)
	Multifidus	40.7 ± 3.4 (12)		43.9 ± 3.7 (12)		(24)
	Erector spinae	39.6 ± 2.8 (12)		41.8 ± 2.3 (12)		(24)

*MF + ES* multifidus and erector spinae combined, *n* total sample size

^a^Sample size of 100 assumed in simulation

More specifically, we created samples of *n* = 1603 (disk degeneration), *n* = 613 (disk signal loss), *n* = 572 (disk height loss), *n* = 430 (disk bulge), *n* = 1408 (disk protrusion), *n* = 996 (annular fissure), *n* = 849 (facet degeneration), and *n* = 284 (spondylolisthesis). Subjects within each sample were distributed across the four age groups of 20–50s according to the sample of subjects reported by Brinjikji et al. [3] in the respective age groups; reported prevalence of degeneration per age group (%) was then used to assign corresponding proportions of 1 (degenerative) and 0 (not degenerative) to subjects within each age group. For example, synthetic data for disk degeneration comprised a total sample of 1603 subjects (273 + 604 + 415 + 311). In age group 20, 101 of 273 subjects were assigned values 1 (37 % degenerative) and 172 subjects were assigned values 0 (63 % not degenerative). Similarly, 314 of 604 (52 %), 282 of 415 (68 %) and 294 of 311 (80 %) subjects in age groups 30, 40 and 50 respectively were classified as having a disk degeneration (Table 1). Logistic regression analyses were then used to estimate prevalence rates from the synthetic data and to determine the marginal effects of age together with CIs for each degenerative feature. For two degenerative features (facet degeneration and spondylolisthesis), no information on the number of subjects in the age-groups 20–29 and 30–39 was available; we assumed a sample size of 100 in each of these two groups. By way of justification, this sample size is roughly twice the size of any reported age-specific sample in the spondylolisthesis group, and of four out of five reported age-specific samples in the facet degeneration group. This yielded CIs that were narrower than they would have been had we assumed sample sizes similar to the reported ones. Consequently, choosing higher sample sizes made it more challenging to demonstrate that marginal effects of age were not different between end-points, i.e. showed a similar rate of decline.

**Fig. 1:**
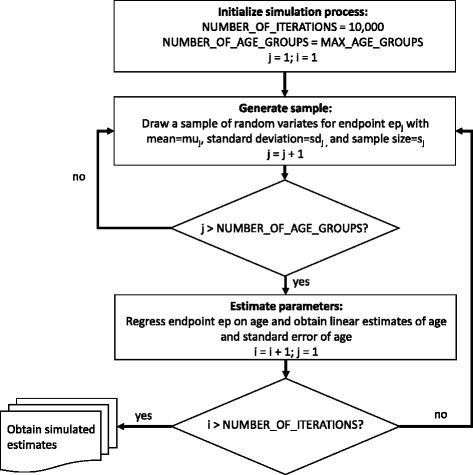
Flow chart of Monte Carlo Simulation process for synthetic Crawford et al. [11] and Valentin et al. [12] data. Footnote: epj corresponds to a random sample of endpoint variates in the j-th age group. muj corresponds to the mean value of the endpoint in the j-th age group. sdj corresponds to the standard deviation of the endpoint in the j-th age group. sj corresponds to the sample size of the endpoint in the j-th age group

Similarly, data from Valentin et al. [12] and Crawford et al. [11] were simulated from information on their published age-aggregated data (Table 1). The original age- and sex-specific sample sizes, means and standard deviations for each muscle’s FI% were used in a Monte Carlo simulation where 10,000 samples of age-specific normal random variates with sample size, mean and standard deviation were drawn equivalent to the original studies. General linear regression models (Gaussian) were then used to estimate marginal effects of age with corresponding CIs. Figure 1 depicts the general flow of the simulation for each endpoint of the Crawford/Valentin muscle studies.

For the Crawford study [11], we created samples of *n* = 80 subjects for each endpoint (i.e. MF + ES). Subjects within each sample were distributed across the four age groups 20–29 to 50–60 according to the sample of subjects reported by Crawford et al. [11] in each age group (*n* = 20) and reported means and standard deviations within each age group (Table 1) were used to create corresponding normal random variates using Stata’s *rnormal* command. Similarly, we created samples of *n* = 24 subjects for each endpoint in the Valentin study [12] (psoas, MF + ES) and subjects within each sample were distributed across the two age groups 18–25 and 46–60 according to the sample of subjects reported by Valentin et al. [12] in each age group (*n*–12). Again, reported means and standard deviations within each age group (Table 1) were used to create corresponding normal random variates. For each endpoint reported in the Crawford [11] and Valentin [12] studies, the process of data generation and a corresponding regression analysis was repeated 10,000 times in order reduce the effect of noise in the simulated data.

For example, the total Crawford MF + ES sample of 80 subjects (20 + 20 + 20 + 20) in Table 1 was simulated by generating 20 normal random MF + ES variates with mean 12.4 and standard deviation 3.7 for age group 20–29 using the Stata *rnormal (12.4, 3.7)* command. Similarly, random MF + ES variates with mean (standard deviation) 15.9 (5.0), 15.7 (4.5) and 18.1 (7.8) were generated for the remaining age groups 30–39, 40–49, 50–60. The generated MF + ES variates were then regressed on age. The MF + ES simulation process, meaning sample generation and regression, was iterated 10,000 times. Where appropriate, a similar simulation procedure was used to estimate gender–specific rates of decline.

Likelihood ratio test was used to make comparison between saturated models using original age-group factor variables, with models employing a linear age predictor derived from the mid-points of the original age-groups. All tests showed that the linearity assumption was not violated. Hence, we report marginal effects of a 1-year change in age (and CIs) for all imaging-identified features of spinal degeneration, including increasing FI in paravertebral muscles, marginal effects per year were used to depict a relative rate of decline per variable.

## Results

**Fig. 2:**
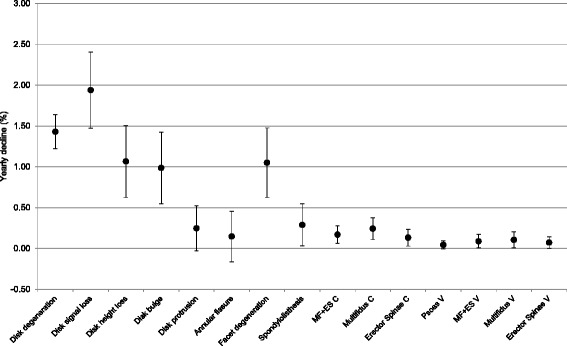
Yearly decline (%) with 95 % confidence interval whiskers for eight degenerative imaging features of the spinal column as derived from Brinjikji et al. [3], alongside paravertebral muscle decline based on Crawford et al. [11] (C) and Valentin et al. [12] (V)

Figure 2 presents our simulated relative rates of decline for eight spinal column degenerative features based on Brinjikji et al. [3], alongside lumbar paravertebral muscle FI% (MF, ES, Psoas and MF + ES combined) based on Crawford et al. (C; [11]) and Valentin et al (V; [12]). Decline in MF + ES (Crawford 0.17 %/year, CI:0.06–0.28; Valentin 0.09 %/year, CI:0.01–0.17), MF (Crawford 0.24 %/year, CI:0.11–0.37; Valentin 0.11 %/year, CI:0.01–0.21), ES (Crawford 0.13 %/year, CI:0.03–0.23; Valentin 0.07 %/year, CI:0.00–0.14) and PS (Valentin 0.04 %/year, CI: −0.01–0.09) occur at similarly slow rates to disk protrusion (0.25 %/year, CI: −0.02–0.52), annular fissure (0.15 %/year, CI: −0.16–0.46) and spondylolisthesis (0.29 %/year, CI:0.03–0.55). Disk signal loss declined fastest at 1.94 %/year (CI:1.47–2.41); its lower boundary of the CI was higher than each variable’s upper boundary except disk degeneration and disk height loss. In descending rate, disk signal loss, disk degeneration, disk height loss, facet degeneration and bulging disk’s lower CI boundaries were higher than upper boundaries of each of the other variables (Table 2). While CIs were overlapping, MF showed a trend for faster decline than ES in both paravertebral muscle studies, while PS had the lowest rate of decline of all parameters. Trends for slower MF decline (women 0.18 %/year, CI: −0.03–0.39; men 0.30 %/year, CI:0.16–0.44) and faster ES decline (women 0.15 %/year, CI: −0.01–0.31; men 0.11 %/year, CI:0.04–0.14) were shown in women (Table 3).

**Table 2:** Regression estimates for yearly decline with synthetic Brinjikji data

Endpoint	*n*	b(age)	se(b)	95 % CI
Disk degeneration	1603	1.429	0.106	1.221–1.637
Disk signal loss	613	1.939	0.238	1.472–2.405
Disk height loss	572	1.065	0.224	0.626–1.505
Disk bulge	430	0.985	0.224	0.545–1.425
Disk protrusion	1408	0.247	0.140	−0.027–0.522
Annular fissure	996	0.146	0.157	−0.162–0.455
Facet degeneration	849	1.050	0.218	0.624–1.477
Spondylolisthesis	284	0.288	0.131	0.032–0.544

*b(age)* regression estimate of yearly decline per age year, *se(b)* standard error of b(age), *n* sample size, *CI* 95 % confidence interval

**Table 3:** Regression estimates for yearly decline obtained from Monte Carlo simulations (synthetic Crawford et al. (C)/Valentin et al (V) data) including data for women and men from the Crawford et al. study

Endpoint	Variable	Iteration	*n*	Mean	Std. Dev.	Min	Max
MF + ES C (women)	b(age)	10000	40	0.162	0.099	−0.222	0.502
	se(b)	10000	40	0.087	0.013	0.046	0.142
Multifidus C (women)	b(age)	10000	40	0.185	0.114	−0.341	0.632
	se(b)	10000	40	0.105	0.014	0.057	0.159
Erector Spinae C (women)	b(age)	10000	40	0.153	0.090	−0.183	0.486
	se(b)	10000	40	0.079	0.013	0.040	0.133
MF + ES C (men)	b(age)	10000	40	0.175	0.044	−0.005	0.328
	se(b)	10000	40	0.048	0.006	0.027	0.077
Multifidus C (men)	b(age)	10000	40	0.299	0.063	0.077	0.544
	se(b)	10000	40	0.070	0.009	0.040	0.102
Erector Spinae C (men)	b(age)	10000	40	0.113	0.032	−0.001	0.232
	se(b)	10000	40	0.035	0.005	0.021	0.055
MF + ES C	b(age)	10000	80	0.169	0.060	−0.072	0.370
	se(b)	10000	80	0.055	0.005	0.037	0.080
Mulitfidus C	b(age)	10000	80	0.244	0.071	−0.036	0.509
	se(b)	10000	80	0.068	0.006	0.044	0.094
Erector Spinae C	b(age)	10000	80	0.132	0.059	−0.088	0.435
	se(b)	10000	80	0.051	0.005	0.036	0.070
Psoas V	b(age)	10000	24	0.044	0.024	−0.048	0.126
	se(b)	10000	24	0.024	0.004	0.012	0.041
Multifidus V	b(age)	10000	24	0.105	0.048	−0.064	0.297
	se(b)	10000	24	0.048	0.007	0.025	0.074
Erector Spinae V	b(age)	10000	24	0.072	0.034	−0.088	0.208
	se(b)	10000	24	0.034	0.005	0.017	0.055
MF + ES V	b(age)	10000	24	0.088	0.040	−0.061	0.239
	se(b)	10000	24	0.040	0.006	0.018	0.062

*b(age)* regression estimate of yearly decline per age year, *se(b)* standard error of b(age), *Iteration* number of iterations in the Monte Carlo simulation, *n* sample size, *Std. Dev.* standard deviation

Tables 2 and 3 give additional and more detailed information on the estimated relative decline rates based on synthetic data and Monte Carlo simulation, including number of iterations, sample size, range and standard errors.

## Discussion

Our study reports a novel method for comparing yearly rate of decline of accepted features of spinal degeneration as determined through imaging studies involving asymptomatic participants. Providing reference values for normative rate of degeneration that includes all structures of the spinal column including muscle represents a new direction for examining spinal tissues, and may have theranostic value in managing the effects of both ageing and LBP.

While muscle tissues are alluded to in the degenerative cascade [13, 30], literature to date has focused on the natural history of degeneration of the vertebra and disks for describing age-aggregated decline of the spinal column. The yearly rate of increasing paravertebral muscle fat content determined in our study was low and in agreement in both muscle studies used, which suggests a relatively slow decline to lumbar muscle quality into healthy adulthood. Whether this trend continues into older age (>60 years) for both genders, and other regional muscles, requires further investigation.

Two population-based studies report longitudinal change to paravertebral muscle quality determined from MRI in participants aged 40 plus [15, 31] and offer a comparison for actual decline to our simulated data. Hebert et al. [31] reported 28.8 % MF fat content at age 40, 28.7 % at 45, and 31.6 % at 49 years of age for their group. While these values indicate a non-linear increase in fat content, the yearly rate of decline calculated between 40 and 49 age time-points is calculated to be 0.31 %. This rate is slightly higher than shown for MF in our two studies (Crawford = 0.24 % and Valentin = 0.11 %), and may be explained by their sample including a proportion of individuals with LBP and/or older age. Fortin et al. [15] reported reduction of functional cross-sectional area (FCSA; fat-free muscle tissue) for MF/ES of 29/42 % at L3/4, and 37/78 % at L5/S1 over their 15 year follow-up of male twins. Assuming linear decline, this suggests yearly rates of 1.9/2.8 % and 2.5/5.2 %, respectively, which are higher again and may reflect their older average age, sex differences [11], a partly symptomatic sample, and/or the differing variable. Interestingly, Fortin et al. [15] reported FCSA/CSA percent reduction for MF/ES to be 0.21/0.30 % and 0.21/0.40 % at L3/4 and L5/S1, respectively. Whether this variable relates best to change to FI requires further substantiation in comparative studies. However, that two population cohorts showed faster paravertebral muscle decline than for the asymptomatic cohorts used in our modelling, suggests potential for differences in rate secondary to painful (or possibly non-painful) symptoms that at least warrants further investigation. Furthermore, the trends for rate differences noted between paravertebral muscles in ours and the Fortin et al. [15] study indicate a non-uniform decline between muscles that may be important in understanding the etiology of FI and any implications for targeted (and likely muscle-differential) therapies.

The relative rate of increasing fat content in paravertebral muscles compared best with that of disk protrusion, spondylolisthesis and annular fissure. While caution should be exercised in making comments regarding common causation between these features, we postulate that an etiological relationship exists. Similar decline rates for spondylolisthesis and muscle quality can be rationalized on a neuro-mechanical basis. Paravertebral muscle denervation is present in asymptomatic individuals [32], with MF purported to be morphologically susceptible to effects of neural stretching after disk height loss and subsequent listhesis resulting in asymptomatic denervation [33]. Kalichman et al. [6] and Teichtahl et al. [9] reported a relationship between disk height loss and paravertebral muscle fat in patients, with the former also showing correlation to spondylolisthesis. Disuse-related muscle atrophy relates to deconditioning, local tensile unload and altered muscle recruitment [4, 34, 35], which Hodges et al. [28] purported was based on changes secondary to structural remodeling rather than atrophy. It seems probable that the faster declining loss of disk height shown in our study precedes declining muscle quality and spondylolisthesis within the normative ageing cascade. Furthermore, we speculate that our results showing descending rates of decline starting from disk signal loss, to disk degeneration, then disk height loss, facet degeneration and disk bulge, preceding disk protrusion, annular fissure, spondylolisthesis and paravertebral muscle tissue decline, are a representation of Kirkaldy-Willis et al.’s [30] degenerative cascade. Longitudinal investigations are required to substantiate this.

The relationship between muscle decline and disk protrusion or annular fissure is difficult to explain without details describing criteria determining each feature from the primary sources referenced by Brinjikji et al. [3], particularly in light of bulging disks being included as a separate category. Both disk protrusion and annular fissure predominate in the posterolateral and posterior disk, respectively and represent disruption to the nociceptive annular layers [36]. Whether the proximity of the posterior disk to the paravertebral muscle tissues and likelihood for exposure to common inflammatory mediators has a bearing on any etiological association to degenerative decline is speculation. However, several animal studies from Hodges and colleagues [28, 35, 37] describe rapid atrophy or re-modelling after experimental disk injury, so an association probably exists.

Age-related change to skeletal muscle quality differs between sexes, wherein, men lose more muscle with ageing, yet women suffer greater functional consequences [24]. Our trend revealing lumbar MF declining faster and ES slower in men than women may reflect sex-dependent degeneration of paravertebral muscle quality whose influence is likely multifactorial. Furthermore, the kinematics of MF and ES based on their different morphology and architecture may point to a need for differential therapeutic strategies in optimizing muscle quality and function.

Comparisons between both muscle studies we used showed consistently lower decline rates in the Valentin et al. [12] parameters, despite reporting higher fat content compared to the Crawford et al. [11] study; sample differences including demographics and activity levels offer a probable rationale for the disparity. While our study references asymptomatic cohorts, we cannot assume that samples included lifetime pain-free subjects, and as such, previous common (or ongoing and underlying) inflammatory mechanisms to decline cannot be discounted.

Our study should be interpreted in light of its limitations. The cross-sectional nature limits its generalizability wherein concordance with secular changes cannot be assumed. Longitudinal studies that concurrently examine degenerative features of the spinal column and paravertebral musculature would offer improvement but are unrealistic in determining rate of decline over the wide age-range described here. Examining normative imaging datasets derived using advanced sequencing like proton density fat-fraction and Dixon fat/water MRI would be warranted in enabling concurrent visualization of both spinal column and adjacent musculature [16–18]. While we consider our simulation design to be statistically sound, drawing comparisons between prevalence rates for spinal degenerative features and actual paravertebral muscle FI is a new approach in determining rate of decline. The robustness of this method might be best tested with comparisons to longitudinal studies that employ methods able to reliably detect serial yearly changes of the small magnitude defined by our study. This is not limited to the lumbar spine region.

## Conclusions

In conclusion, lumbar paravertebral muscle degeneration as defined by increasing FI appears to occur relatively slowly in asymptomatic adults aged 18 to 60 years in comparison to degeneration of the spinal column. Muscle decline warrants inclusion as a feature of the normative degenerative cascade. Further investigation is necessary to determine the functional significance of the rate of decline of paravertebral muscle tissues, and by comparison to people with LBP and other musculoskeletal disease.

## Abbreviations

CI, 95 % confidence intervals; ES, erector spinae; FI, fatty infiltration; LBP, low back pain; MF, multifidus; MF + ES, multifidus & erector spinae combined; PS, psoas; Theranostic, convergence of terms therapeutic, diagnostic and prognostic
